# Object and tool manipulation diversity in an urban capuchin monkey (*Sapajus libidinosus*) group in Brasília National park

**DOI:** 10.1007/s10329-026-01242-7

**Published:** 2026-03-07

**Authors:** Túlio Costa Lousa, Murilo Reis Camargo, Ricardo Vasquez Mota, Francisco Dyonísio Cardoso Mendes

**Affiliations:** 1 Departament of Experimental Psycology, Universidade de São Paulo, São Paulo, 05508-030 Brazil; 2 Department of Basic Psychological Processes, Institute of Psychology, University of Brasília, Brasília, Brazil; 3 FacUnicaps, Goiânia, 74535-040 GO Brazil; 4 Centro Universitário IESB, Ceilândia Norte QNN 31, Brasilia, 72225-315 Federal District Brazil

**Keywords:** Proto-tool use, Simple manipulation, Anthropogenic environment, Urban ecology, Food acquisition rate

## Abstract

Capuchin monkeys are highly skilled primates recognized for their diverse object manipulation, a crucial evolutionary trait. This study explores the influence of the urban, artificial setting of Brasília National Park (BNP)on capuchin monkeys’ object manipulation behaviors, aiming to offer more systematic data on this activity within urban Cerrado environments. Object manipulation data were systematically gathered using the “all occurrences” method and video analysis, examining the object, action, and target of each manipulation, along with contextual elements such as date, actor, time, function, type, and food acquisition. Tool use involves modifying an object, substrate, food, another individual, or the self; proto-tool use consists of hitting an object or food against a fixed substrate; simple manipulation refers to handling or carrying a single object without contact. Statistical tests conducted in SPSS 26.0 and R console, including GLMM, binary logistic regression, and Spearman correlation, evaluated individual differences in manipulation involvement, predicted acquisition rates, and assessed links between simpler manipulations and tool use, excluding unclear actor data from statistical but not descriptive analysis. The artificial environment of BNP notably influenced the monkeys’ manipulation behaviors, introducing novel anthropogenic materials and altering their techniques relative to natural populations. While overall manipulation frequency varied by sex and age (with males and juveniles manipulating more), specific manipulation types did not show such variation, and tool use correlated with simpler manipulations, supporting a learning-based progression Unexpectedly, tool use yielded lower rates of food acquisition than other manipulation types. Furthermore, food acquisition through tool use was more frequent among males, possibly attributable to the dominant male’s lack of tool use, which did not provide a reliable model for this behavior.

**Supplementary Information:**

The online version contains supplementary material available at 10.1007/s10329-026-01242-7.

The proportion of people living in cities is increasing, leading to an acceleration of the urbanization process (United Nations 2024). Urbanization has resulted in permanent alterations to the planet, rapidly modifying environments (Ruas et al. 2022), and negatively influencing primate species richness (Torres-Romero et al. 2023). The urban environment increases the risk of primate mortality due to roadkills (Praill et al. 2023), diseases (Devaux et al. 2019), and electrocutions (Barros et al. 2025). Despite these risks, some primate species are capable of thriving in the urban environment, taking advantage of the available anthropogenic resources (Thacher et al. 2023).

This process of urbanization brings wild primates into closer proximity with humans, compelling them to utilize areas increasingly near densely inhabited regions (Garcia de la Chica et al. 2023). Wild primates are driven to seek food possessed by humans (e.g., Thatcher et al. 2021) and to foraging on human refuse (Lousa et al. 2022; Anca and Wallis 2024; Albuquerque Freire et al. 2025). This close proximity to humans and anthropogenic objects may modify the way primates interact with the world and may introduce novel cognitive challenges for the animal (Lee and Thornton 2021; Pal et al. 2022).

Urbanization can modify the behavior of primates (Kaburu et al. 2019), including the manipulative behavior (Roncero et al. 2023). The direction of modification resulting from urbanization can manifest either as a decrease in manipulative complexity (Roncero et al. 2023 in capuchin monkeys) or as an increase in frequency (Jaman and Huffman 2013 in Rhesus macaques), variability (Mangalam and Singh 2013 in bonnet macaques) and sophistication of the techniques (Pal et al. 2022 in bonnet macaques). Furthermore, Dhananjaya et al. (2022) reported that, across three Old World monkey species, there was an observed increase in the reliance on hands compared to the mouth during food acquisition in groups inhabiting more urbanized areas.

Capuchin monkeys (*Sapajus* genus) are primates renowned for their exceptional manipulative abilities, a trait considered a significant evolutionary characteristic across the entire primate order (Truppa et al. 2019). In their natural habitats, these monkeys exhibit a diverse range of object manipulations. They engage in tool use, such as using stones for cracking palm nuts (Fragaszy et al. 2023), throwing objects at conspecifics (Falótico and Ottoni 2013), using stones for digging, and employing sticks for probing (Falótico et al. 2017, 2021; Mannu and Ottoni 2009). They may also use proto-tools, such as rubbing an object on the ground, or perform simple manipulations, such as holding an object directly with their hands (Resende et al. 2008).

In capuchin monkeys, males manipulate more frequently and are more effective in tool use them females, especially regarding nut-cracking of hard-resistant targets or using stick tools (Falótico et al. 2021; Spagnoletti et al. 2011; Falótico and Ottoni 2014; Moura and Lee 2010; Cardoso and Ottoni 2016). This heightened effectiveness might be attributable to the larger body size of males, making them more effective in using percussive tools for cracking (Spagnoletti et al. 2011; Reader and Laland 2001). Alternatively, this sex difference could also be explained by developmental factors, specifically, where males may be more attracted to some manipulative stimuli during development (Falótico et al. 2021). Furthermore, energetic factors may play a role, as females must allocate their effort toward reproduction and offspring care, thus potentially avoiding the risk of eventual failure in more complex manipulations (Spagnoletti et al. 2011).

Within Brasília National Park (BNP), located within the urban area of Brasília, Brazil, palm nut cracking by capuchin monkeys (*Sapajus libidinosus*) in a Cerrado environment was previously documented (Waga et al. 2006), alongside other forms of object manipulation (Mota 2018). In the BNP, routine contact between capuchin monkeys and humans is common, leading to the acquisition of human-related resources (Camargo et al. 2024; Lousa et al. 2024). The study group intensified this human proximity by spending a significant amount of time in the public visitation site known as the *Pedreira* pool. This spatial overlap may have altered the repertoire of manipulated objects due to the enhanced availability of anthropogenic items. A notable characteristic of object manipulation in BNP was the use of artificial rocks as hammers in cracking activities.

Furthermore, the findings from the BNP study by Waga et al. (2006), while relevant, are considered preliminary due to the limited observation time and low number of recorded events. Previously, other studies on tool use in urban capuchin monkeys (e.g., Ottoni and Mannu 2001; Ottoni et al. 2005; Resende et al. 2008; Coelho et al. 2015; Corat et al. 2015; Aguiar et al. 2014; Gutierres et al. 2025) were conducted in different biomes, with distinct capuchin species, and under environmental conditions that varied from those observed in the BNP.

Despite existing research, a consensus on the proximate factors influencing object manipulation and stone tool use for nut-cracking among urban capuchin monkeys in the Cerrado remains elusive due to insufficient data. The impact of human activities on manipulative behaviors is also not fully understood. Our primary objectives were twofold: first, to enhance the systematic data on these behaviors; and second, to analyze how anthropogenic factors influence the manipulative behaviors of the monkeys in BNP.

Based on the significant human influence in the BNP we developed several predictions. We first predicted that artificial objects and food would be manipulated more often than natural items. This prediction stems from the observation that, in urban environments, artificial objects are typically discarded in refuse sites (garbage) and serve as a readily accessible source of artificial food items, often being clustered and easily reachable by capuchin monkeys (Camargo et al. 2024). Furthermore, given the high degree of environmental artificiality within BNP, we predicted that artificial objects, such as paved stones and packaging remnants, would be more readily available for the animals’ manipulative behaviors. Consistent with findings from other urban primate populations, we also predicted that individuals with more contact with refuse or higher levels of human interaction would exhibit an increased diversity of manipulation and tool-use techniques (Mangalam and Singh 2013; Jaman and Huffman 2013; Pal et al. 2022). Additionally, we predicted that tool use would be a more effective strategy than simpler manipulations, a prediction premised on the assumption that simpler manipulations serve as a learning process for complex tool use (Koops et al. 2015). Finally, we predicted that males would engage in object manipulation more frequently than females, as this is the typical pattern observed for the genus *Sapajus*, especially regarding nut-cracking of hard-resistant targets or using stick tools (Falótico et al. 2021; Spagnoletti et al. 2011; Falótico and Ottoni 2014; Moura and Lee 2010; Cardoso and Ottoni 2016).

## Methodology

### Subjects and study site

**Fig. 1:**
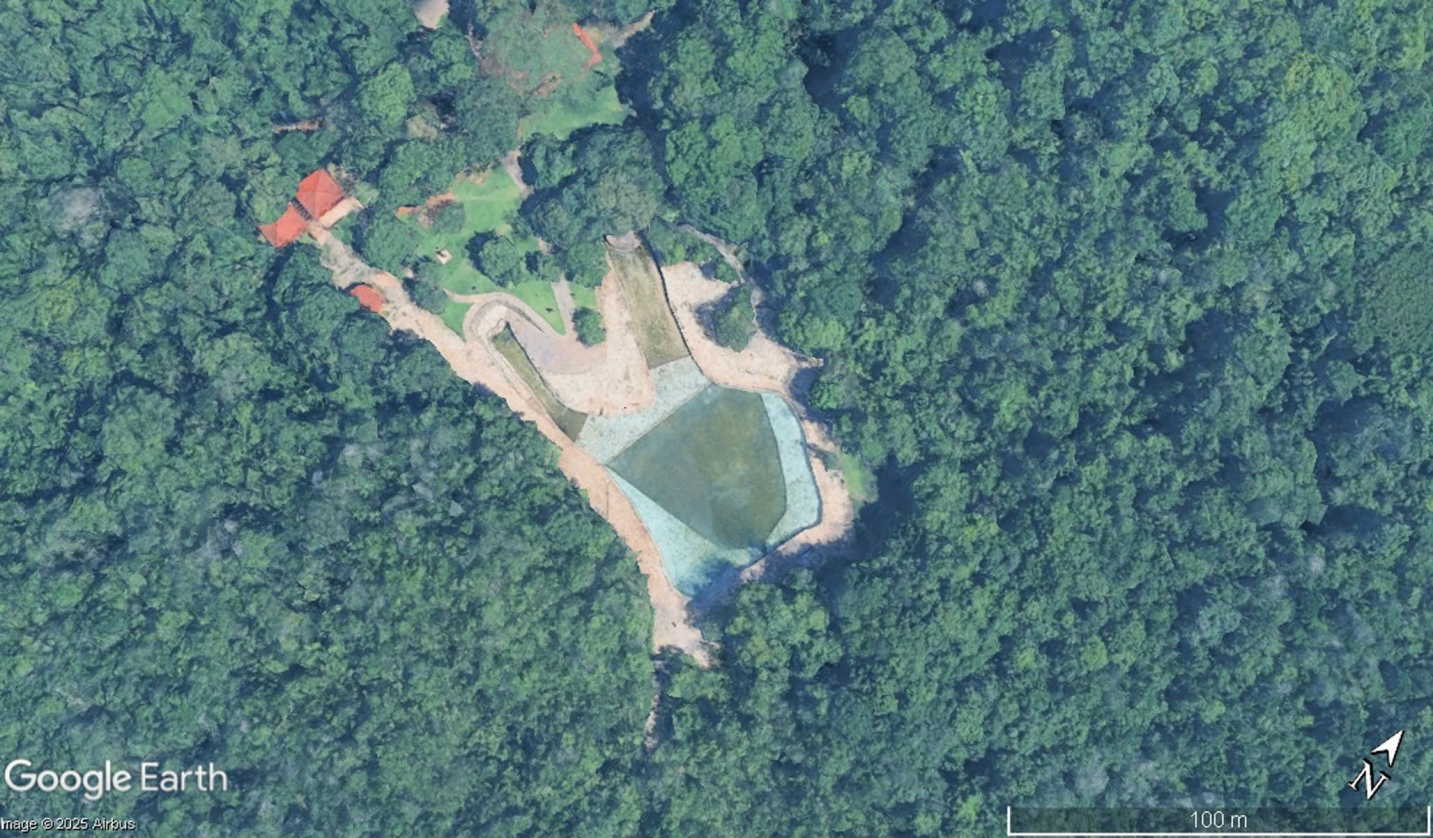
The running water pool at Brasília National Park (*Pedreira* Pool), the primary location for anthropogenic object manipulation

The study was conducted in Brasília National Park (15º43’S, 47º55’W). The BNP is the largest conservation unit in the Federal District, with an area of 46,230 hectares. Its vegetation is representative of the Cerrado biome, the Brazilian Savanna, with phytophysiognomies varying between almost pure, open grasslands and areas of low forest with approximately 50% coverage (Horowirtz et al. 2013). The region’s climate is tropical seasonal, with a rainy season between October and March and a dry season between April and September (Sacramento et al. 2017). The park contains two natural swimming pools, usually open to the public, which attract many visitors (Camargo et al. 2024; Sabbatini et al. 2006) (Fig. 1).

We studied a capuchin monkey group that regularly visits one of these natural pools (*Pedreira* pool) and is fed daily by visitors. This provisioning leads to numerous interactions, sometimes resulting in conflicts between the capuchin monkeys and humans (Camargo et al. 2024; Lousa et al. 2024). The group’s size varied between 12 and 14 individuals during the study due to two births, comprising 5 adults (3 males: 2 females), 6 juveniles (3 males, 1 female and 2 unidentified), and 3 infants. Prior to this study, we confirmed the observation by Waga et al. (2006) that some individuals in the group use tools to crack nuts.

**Fig. 2:**
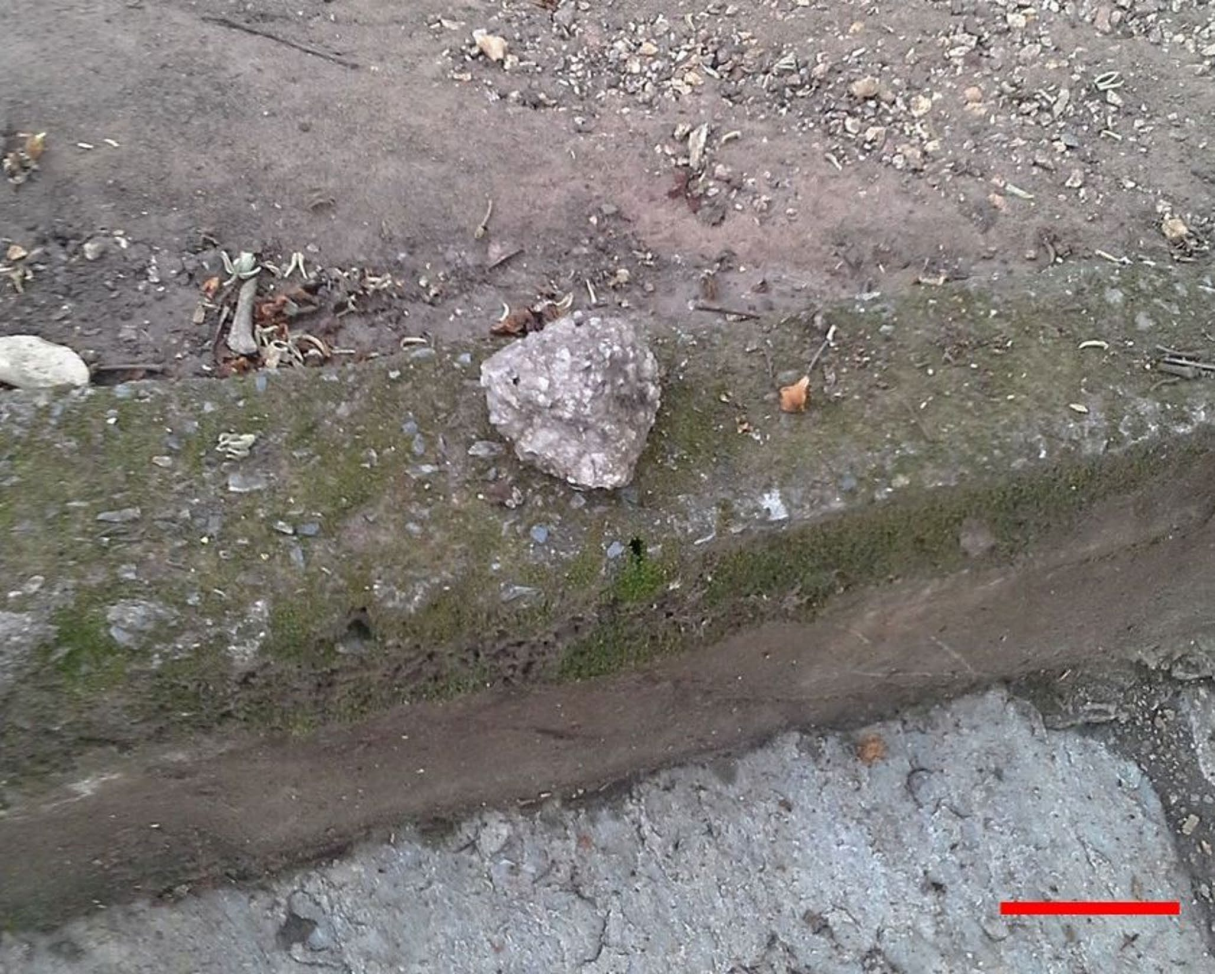
A nut-cracking site where construction debris served as the hammer and the pavement acted as the anvil. Scale bar 10 cm

The majority of manipulative activities occurred in the *Pedreira* Pool area (Fig. 1), which contained structures built from concrete, stone, and wood. The forest adjacent to the pool contained construction debris, including brick blocks and slate stones (Fig. 2). These materials were transported to locations with human presence, either by the monkeys or by visitors.

### Data collection

Systematic data collection took place from July 2016 to December 2017. Observations were conducted four to six days a week, alternating between mornings and afternoons, accumulating 942 h of field effort. To quantify the frequency of object manipulations and analyze the form and context in which they occurred, the “all occurrences” method was used (Altmann 1974). Data was collected both through in situ note-taking on recording sheets and via video recordings analyzed subsequently using the same protocol.

**Fig. 3:**
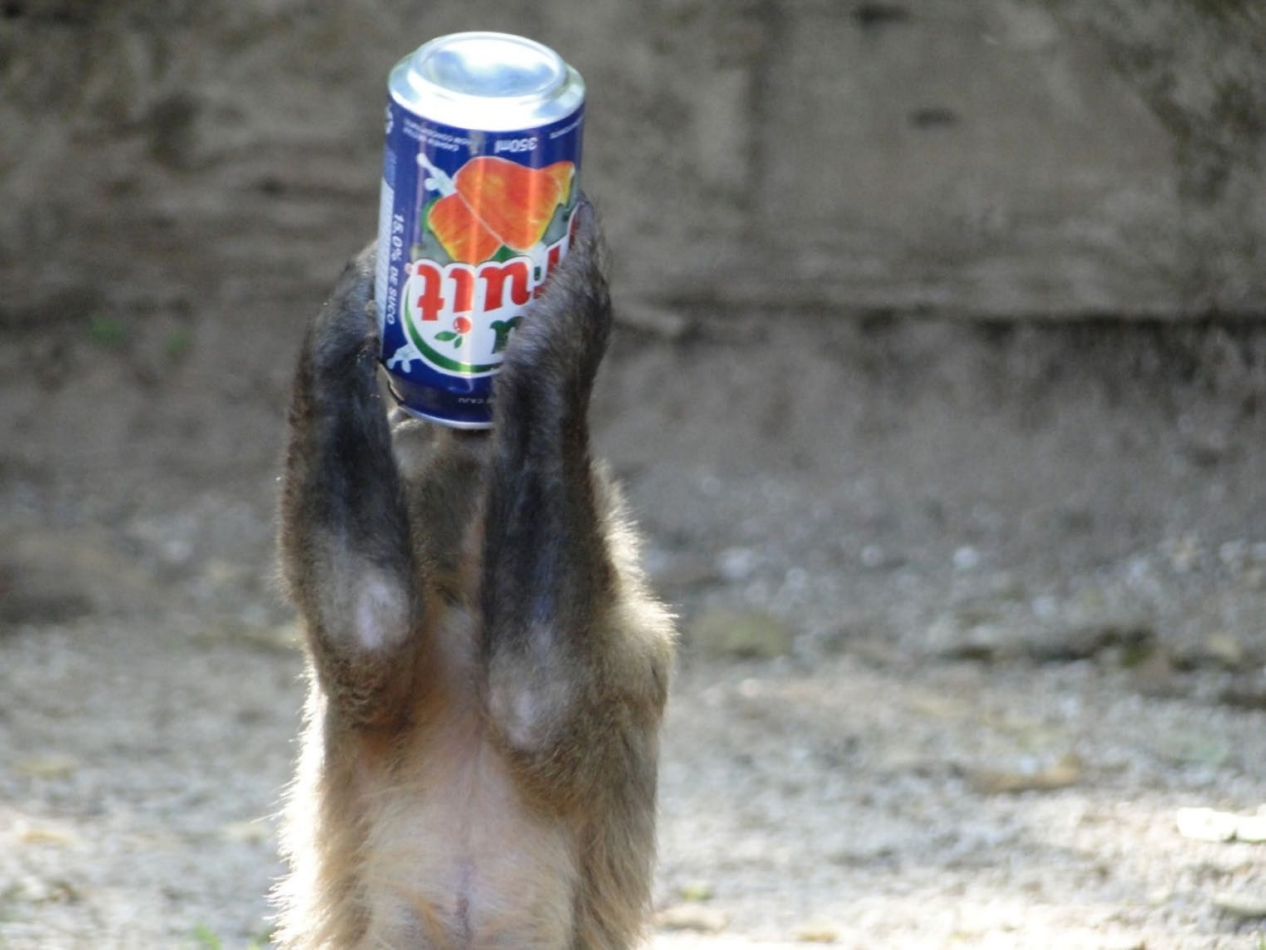
Capuchin monkey manipulating an anthropogenic object (juice can), illustrating the unpredictability and availability of human-derived objects at the site

To understand the manipulation events, we focused on identifying the “object” being used, the “action” performed with it, and the “target” of that action (e.g., a stone used to hit the ground). This recording method was chosen due to the unpredictability of the objects used (Fig. 2), as well as the actions performed and the targets hit, which made a prior definition of specific categories impossible. Thus, the filling of these items in the ethogram remained, initially, open and according to the behavior performed by the animal. This method was primarily based on the effect caused by the use of the object, avoiding possible subjective interpretations regarding the actions performed. Additionally, the following data were noted: date, actor, start and end of observation per day, time of manipulation, function/context of use (e.g., palm nut cracking, simple handling), type of manipulation (Table 1), acquisition of the food item (obtaining food at the end of the food process, considering only food-related manipulations), and, if necessary, a description of the activity performed (Fig. 3).

**Table 1:** Classification of object manipulations (based on Shumaker et al. 2024; resende et al. 2008; Parker and Gibson 1977)

Type of manipulation	Definition
Tool	Manipulation of one detached object to modify the shape or position of another object, substrate (fixed or not), food, individual, or part of one’s own body. The use may or may not facilitate or allow access to food or another object. (e.g. raking in one object with another)
Proto-tool	Manipulation of one object or food relative to a fixed substrate (for example, rubbing an object on the ground).
Simple manipulation	Manipulating objects or the substrate directly (e.g. Fig. 3, holding a juice can).

The types of objects, actions, and targets recorded were grouped into categories after data collection. Objects were classified in natural food (fruits foraged in the environment), anthropogenic food (provisioned food), stone, plant parts (twigs and bark), trunk, insects, and artificial objects. Actions were defined based on the effect that manipulation could cause on the target, being divided into hit, scrape, transport, shake, throw, handle, and rub (Table 2). Targets, in turn, were categorized based on the type of effect caused on the item or environment reached, resulting from the action carried out by the subject, and included food, trees and other wooden items (e.g. trunks and posts), stone, soil, plant parts, own body, other animals, and artificial objects.

**Table 2:** Definitions of the actions based on Hayashi (2015) and Torigoe (1985)

Action	Definition
Throw	Launching an object through the air.
Hit	Striking an object with or without holding another object.
Rub	Moving an object against a body part.
Handle	Holding a single object without it touching any other object, surface, food, another individual, or the animal’s own body.
Scrape	Dragging an object across the floor or a wall.
Shake	Moving an object back and forth or in a circular motion.
Transport	Moving from one place to another while carrying an object.

For the context, we followed the same procedure, categorizing based on the overall purpose of the activity. We categorized the observed behaviors by their overall function, resulting in the following contexts: feeding (note that palm nut cracking was a separate category due to its unique movements and technique); occupational, palm nut cracking itself, transport, throwing objects; repellent behavior, and reactions to aversive stimuli (Table 3).

**Table 3:** Definition of the contexts based on Hayashi (2015), Torigoe (1985), Falótico et al. (2007), Verderane et al. (2007), and muricy Filho et al. (2021)

Context	Definition
Feeding	When the animal employs manipulation to obtain food.
Throwing Objects	When an individual actively swings their forelimb to release and propel an object through the air.
Occupational	Activities that don’t seem to have an immediate purpose, like hitting a stone on the ground.
Palm Nut Cracking	Complex tool-use behavior in capuchin monkeys where they use a hammer stone to strike and open nuts against a fixed anvil (like a rock or tree root).
Predation	When the animal manipulates an object to capture prey.
Repellent	Which involved using insects on the body to obtain mite-repelling substances.
Reactions to aversive stimuli	The intense manipulation of objects when a potential threat is present.
Transport	Using an object as a container to help move another item.
Simple Handing	Manipulating objects or the substrate directly

### Data analysis

For statistical analysis, we utilized SPSS 26.0 and the lme4 package in the R console (Bates et al. 2015). A Generalized Linear Mixed Model (GLMM), modeled with a Poisson distribution and a Log link function, was employed to assess how individual characteristics (identity, sex, and age) and the type of manipulation influenced the frequency of participation in these activities. The model’s fixed effects included sex, age, and manipulation type, with the individual monkey’s identity specified as a random effect. To quantify specific differences in participation frequency between the levels of the manipulation type factor, as well as the sex and age factors, Estimated Marginal Means (EMMs) were calculated using the emmeans package (Lenth 2025). The DHARMa package (Hartig 2024) was employed for model diagnostics and validation.

The acquisition of the food item of manipulations was analyzed using binary logistic regression (LR), with acquisition of the food item as the outcome variable and the individual’s sex, age, and the type of manipulation as predictors. To investigate the relationships among these behaviors, we performed a Spearman correlation (*p* < 0.05). This analysis examined the associations between the frequency of proto-tool, simple, and tool manipulations by individuals. Furthermore, it explored the relationships between the frequency of these behaviors and both human interaction (Lousa et al. 2024) and contact with garbage (Camargo et al. 2024; Table 7). We excluded records with unclear actor identity from statistical analyses but included them in the descriptive overview.

### Ethical note and data sharing

Ethical approval for all stages of this research was obtained from the Brasília University Committee on the Ethical Use of Animals, Institute of Biology (protocol 153395/2015), before data collection began. We also secured permission from SISBIO-ICMBIO (protocol 46301-12) to conduct our study within Brasília National Park, a federally protected area.

## Results

### Description of object manipulations

Our observations revealed 406 episodes of object manipulation within the capuchin monkey group. The majority of these involved proto-tools (72.2%), followed by tool use (21.4%), and simple manipulations (6.4%). These behaviors were observed in a range of contexts (Table 2), with feeding (43.35%) and occupational activities (30.08%) being the most prevalent. Notably, we also recorded more complex and less frequent manipulations, including palm nut cracking (17.98%) and one instance of object throwing.

The objects used, as well as the actions performed and the targets hit, also varied greatly (Tables 4 and 5). However, there was a predominance of stone objects, wild fruits, and anthropogenic fruits, of the action ‘hit’, and of targets that were typically trees/wooden items or food. Some of the objects used were of anthropogenic origin, such as plastic bottles and kitchen pots (packaging); these were used to hit and scrape surfaces, transport items, or were transported. The stones used as hammers in cracking, however, were often artificial and obtained in or around the pool area (e.g., pieces of roofing slate or similar material). In terms of actions, several unusual episodes were also documented. These included throwing an object (1), which was directed at another animal, and rubbing insects (1) on the body. Additionally, some behaviors involved artificial objects as targets. One instance involved a bottle cap (1) and another piece of chewing gum (1) being struck with a stone, which resembled a form of “inadequate palm nut cracking.“.

**Table 4:** Categories of object, action, target and context categories present in object manipulations distributed by type of manipulation

Type	Categories	Simple Manipulation	Proto-Tool	Tool	Total
Object	Biscuit	0	1	0	1
	Packaging	1	11	2	14
	Stone	4	102	79	185
	Wood	0	4	0	4
	Anthropogenic Fruit	5	46	2	53
	Branch	4	13	1	18
	Insects	0	0	1	1
	Wild Fruit	12	116	2	130
Action	Throw	0	0	1	1
	Hit	0	277	82	359
	Rub	0	0	1	1
	Handle	8	0	0	8
	Scrape	0	16	1	17
	Shake	2	0	0	2
	Transport	16	0	2	18
Target	None	26	0	0	26
	Food	0	0	76	76
	Tree/Wooden Items	0	191	0	191
	Bark	0	0	2	2
	Artificial Objects	0	0	1	1
	Other Animal	0	0	1	1
	Stone	0	51	6	57
	Own Body	0	0	1	1
Context	Not Identified	0	0	2	2
	Food Processing	0	170	6	176
	Throwing Objects	0	0	1	1
	Simple Handing	8	0	0	8
	Occupational	0	123	2	125
	Palm Nut Cracking (Food Processing)	0	0	73	73
	Predation	2	0	0	2
	Repellent	0	0	1	1
	Transport	16	0	2	18
	Total	26	293	87	406

**Table 5:** Frequency of manipulation of anthropogenic and natural objects

Type	Object	Simple	Proto-tool	Tool
Anthropogenic	Biscuit	0	1	0
	Packaging	1	11	2
	Stone	4	102	79
	Wood	0	4	0
	Anthropogenic Fruit	5	46	2
	Total	10	164	83
Natural	Branch	4	13	1
	Insects	0	0	1
	Wild Fruit	12	116	2
	Total	16	129	4
Grand Total		26	293	87

We observed monkeys carrying artificial plastic objects filled with human food (fruit peels and bones). In two observed instances, the food was already inside the plastic container when we recorded the event. Conversely, in one instance, a monkey first retrieved food from the ground and then placed it within the plastic object for transport. Interestingly, the outcomes differed: in the first two cases, the transported food was consumed, and the plastic object was manipulated further, while in the single latter case, both the food and the plastic object were abandoned without being used.

Most nuts targeted for tool use activities were from palm trees typical of the Cerrado and Caatinga biomes, such as Jerivá (*Syagrus romanzoffiana*), Macaúba (*Acrocomia aculeata*), and Buriti (*Mauritia flexuosa*). In at least thirteen breaking episodes, however, the fruits struck were from species different from those traditionally broken, e.g., Gameleira branca (*Ficus gomelleira*), and various seeds – see Mendes et al. (2015), with juveniles being the actors in eight of these instances.

### Sex/Age differences

**Fig. 4:**
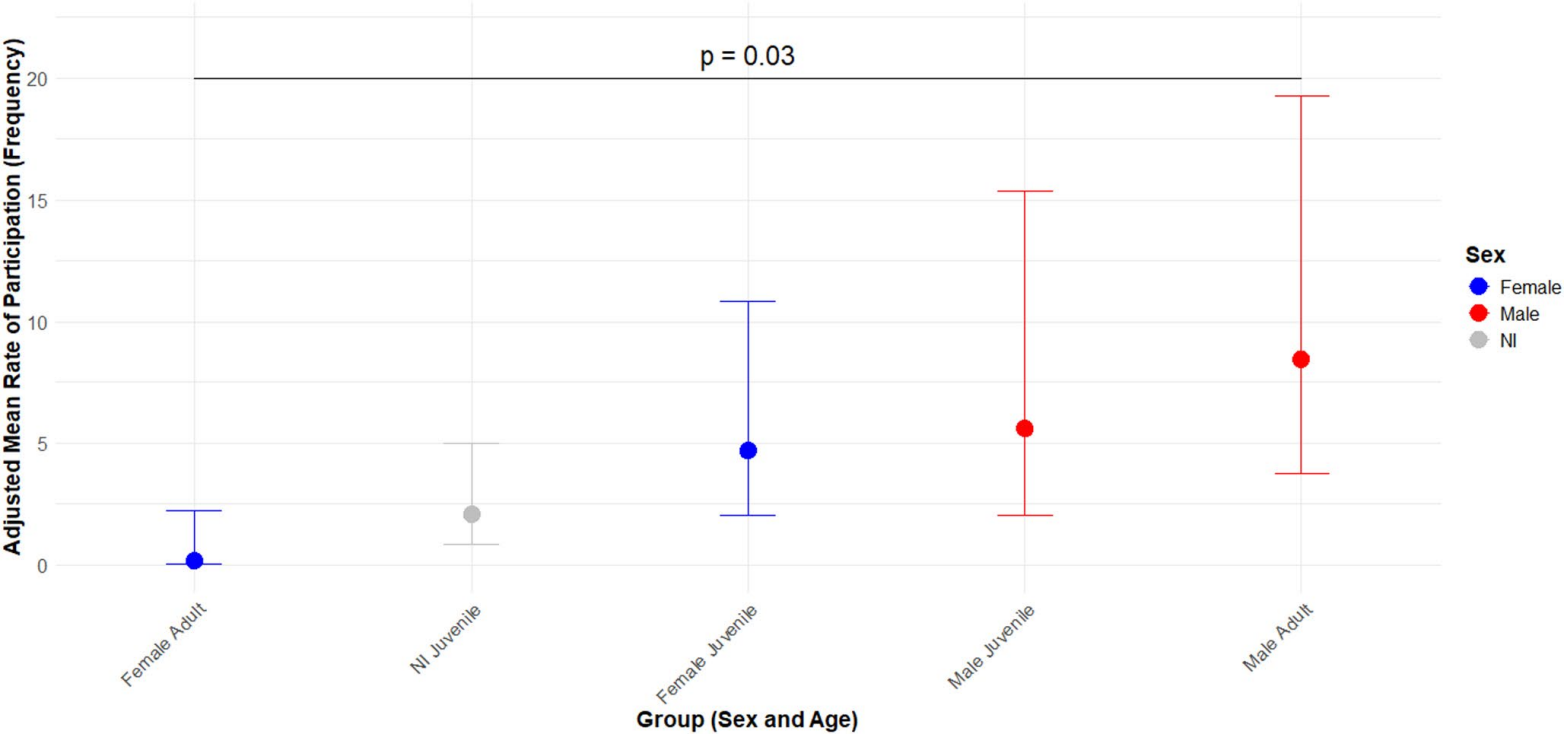
Predicted count of object manipulation events by Age and Sex. Points represent the predicted mean count of manipulation events based on the GLMM, adjusted for “Tool” manipulation type and population-level monkey identification random effect. Error bars indicate the 95% confidence intervals. Age categories include Adult and Juvenile, while Sex categories are Female, Male, and Unidentified

Manipulation events differed across sex and age (Table 5; Fig. 4) categories. Adult Males performed 43.48 times more manipulation events than Adult Females (OR = 0.023, Z = 2.897, *p* = 0.03). An interaction was observed between sex and age: females showed a decreasing trend in the number of manipulations as they aged, while males maintained stable manipulation quantities, with a non-significant increase over time (Table 5).

Proto-tools occurred 2.88 times more frequently than tools (OR = 0.347, Z = 7.954, *p* < 0.001) and 9.43 times more frequently than simple manipulations (OR = 0.106, Z = 10.291, *p* < 0.001). Furthermore, tools occurred 3.26 times more frequently than simple manipulations (OR = 3.261, Z = 4.998, *p* < 0.001) (Table 5).

The Poisson distribution was confirmed as appropriate, as the calculated dispersion parameter (phi = 1.001) indicated no significant overdispersion. Furthermore, the residuals passed the uniformity test (KS test *p* > 0.31) using the DHARMa package, ensuring the model’s robustness and validating its use for inference (Table 6.

**Table 6:** Summary of generalized linear mixed model (GLMM) output

Term	Estimate	Std. Error	z value	*P* value
Sex Male	3.771	1.302	2.897	0.004*
Sex Unidentified	− 0.817	0.614	− 1.331	0.183
Age Juvenile	3.180	1.305	2.438	0.015*
Simple Manipulation	− 1.182	0.238	− 4.959	< 0.001*
Proto-Tool	1.058	0.134	7.893	< 0.001*
Sex Male * Age Juvenile	− 3.593	1.461	− 2.460	0.014*

* *p* < 0.05

## Individual differences

A strong, positive correlation was found between the frequency of tool use and both proto-tool use (*r* = 0.912) and simple manipulations (*r* = 0.889). This finding was supported at the individual level, as six of the seven most frequent actors of proto-tool and simple manipulation were also the most active in palm nut cracking and tool use. Furthermore, moderate positive correlations were observed between simple manipulation and human interaction (*r* = 0.537), as well as between simple manipulation and foraging in garbage (*r* = 0.607). A similar moderate positive correlation (*r* = 0.538) was also found between proto-tool use and foraging in garbage (Table 7).

**Table 7:** Frequency and relative rank position of individuals and regarding human interaction, foraging in garbage, simple manipulation, tool use, and proto-tool use

Monkey ID	Human Interaction	Garbage -	Simple	Proto-Tool	Tool
Mi (AM)	111 (3)	134 (1)	7 (1)	50 (1)	27 (1)
Rc (JM)	27 (6)	29 (7)	3 (3)	23 (4)	14 (2)
Di (AM)	195 (1)	113 (2)	5 (2)	39 (2)	13 (3)
Pe (JF)	8 (10)	15 (9)	3 (3)	30 (3)	9 (4)
Ca (JF)	18 (7)	40 (6)	3 (3)	21 (5)	5 (5)
Jo (IM)	9 (9)	3 (11)	0 (8)	12 (7)	3 (6)
Fo (JM)	10 (8)	19 (8)	1 (6)	13 (6)	2 (7)
Go (JM)	0 (14)	0 (14)	0 (8)	3 (11)	1 (8)
Ra (AM)	67 (4)	40 (5)	1 (6)	7 (9)	1 (9)
An (JU)	2 (11)	2 (12)	0 (8)	0 (13)	0 (10)
Co (AF)	64 (5)	101 (3)	0 (8)	1 (12)	0 (10)
Ct (JU)	1 (12)	2 (13)	0 (8)	0 (14)	0 (10)
Fa (JU)	1 (13)	9 (10)	0 (8)	11 (8)	0 (10)
Ri (AF)	166 (2)	87 (4)	0 (8)	6 (10)	0 (10)

*AM* Adult male, *AF* Adult female, *JM* Juvenile male, *JF* Juvenile female, *JU* Juvenile

## Acquisition of the food item

**Fig. 5:**
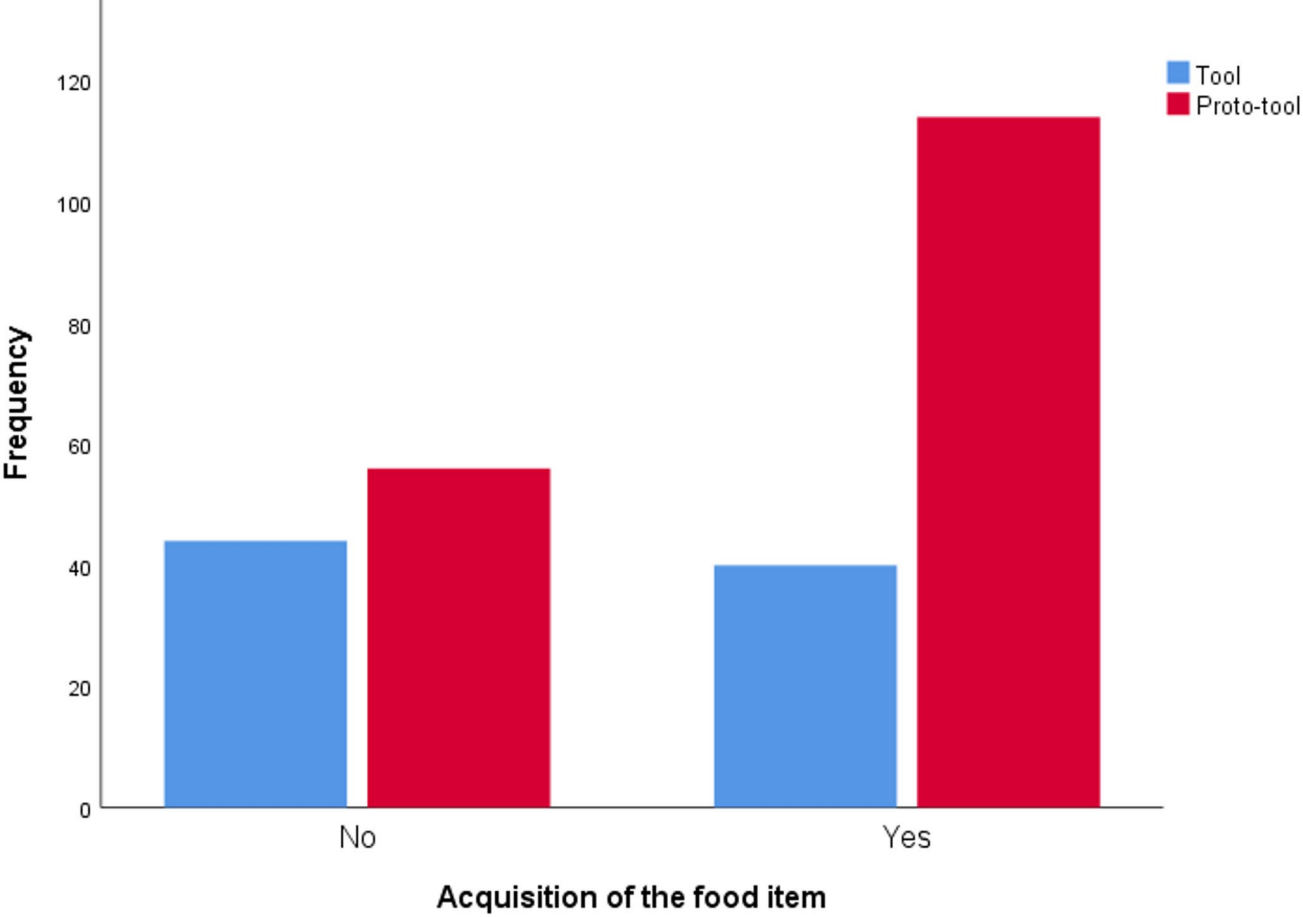
Occurrences by type of food processing in relation to the acquisition of the food item (i.e., obtaining food at the conclusion of the food process event)

Males obtained the food item significantly more often through food processing compared to females (B = 1.341, df = 1, *p* = 0.007, Exp(B) = 3.823). Specifically, males achieved a food acquisition rate of 68.657%, while females achieved an acquisition rate of 62.069%. Furthermore, individuals acquired food more frequently when the food process did not involve tool use (B = − 1.213, df = 1, *p* < 0.001, Exp(B) = 0.297 – Fig. 5), The rate of food obtainment was 67.059% during non-tool use processing (proto-tools) and only 47.619% when tools were employed.

## Discussion

Even though the capuchin monkeys in Brasília National Park (BNP) did not exclusively manipulate artificial items or anthropogenic food, the anthropized environment significantly influenced their manipulative behaviors. The presence of a human-modified environment led to a considerable variety in the categories of objects, actions, and targets, as well as in the contexts of object manipulation. These monkeys had access to visitor-related items not available in entirely natural settings, such as artificial objects (e.g., plastic containers, aluminum cans) and anthropogenic foods. Specifically, the study group utilized artificial objects as hammerstones for activities such as palm nut cracking, a behavior previously described by Waga et al. (2006) and observed in the current study. We also documented these artificial materials being used in other contexts, including striking fixed surfaces, soft fruits, and other artificial objects. Furthermore, while subjects had access to a large amount of anthropogenic food from visitors (Camargo et al. 2024), which they directly ingested, they also incorporated it into their manipulative behaviors, using it as both targets and, at times, as the manipulated objects themselves.

In contrast to the findings of Bender (2024) in Foz do Iguazu, capuchin monkeys in our study manipulated natural items at a higher frequency than artificial items (Table 5). Alson, in congruence with findings from other urban capuchin populations, individuals in the BNP also used construction debris as hammers and anvils for palm nut cracking (Ottoni and Mannu 2001; Ottoni et al. 2005; Resende et al. 2008; Coelho et al. 2015; Corat et al. 2015; Aguiar et al. 2014). The availability of this construction debris may have facilitated an increased frequency of palm nut cracking within this population, as such records are notably scarce for neighboring groups in the BNP (Personal Observation).

In wild populations of capuchin monkeys with spontaneous tool use studied, there is not much diversity in the objects used, with the employment basically of stones and, in some cases, sticks (e.g., Ottoni and Izar 2008). The quantity of actions performed and the contexts in which they occur, however, are very varied and generally complex in terms of topography and functionality (i.e., cognition). In Piauí, for example, the groups were observed using stones to crack nuts, cut, dig, throw at conspecifics (Falótico et al. 2017; Falótico and Ottoni 2013, 2016; Mannu and Ottoni 2009; Fragaszy et al. 2023); sticks were used as probes to access food and branches as devices to reach conspecifics (Falótico et al. 2021; Falótico and Ottoni 2014; Visalbergui et al. 2017). In our research, so many functions for object manipulations were not observed, and regarding tools specifically, the frequency of use was lower. Factors such as terrestriality (Visalbergui et al. 2005; Falótico and Ottoni 2023; Heldstab et al. 2016) and behavioral traditions (Ottoni 2015) would be acting in this sense, with individuals in Piauí having more opportunity to descend to the ground due to low vegetation and flat terrain, and the history of social learning between generations is possibly older, dating, in one of the populations, from the pre-Columbian period (Haslam et al. 2016). Despite this, interesting results were found in our group, such as the use of stones for palm nut cracking, in addition to isolated occurrences of object throwing and the use of objects as containers for transporting food, for example.

As expected, adult males were responsible for the majority of manipulation events. However, when considering the relative distribution of the specific types of manipulation, a similar pattern emerged across sex and age categories, with the highest frequency of events observed in proto-tools, followed by tools, and a smaller number of simple manipulations.

As anticipated, males exhibited a higher overall frequency of object manipulation. However, unlike the findings of Falótico et al. (2021) in capuchin monkeys and Koops et al. (2015) in Chimpanzees, we found no difference in manipulation frequency between immature males and females, with this sex difference only emerging in adulthood (Goldsborough et al. 2024; Reader and Laland 2001).This lack of a sex-based difference in frequency was specific to simple manipulations, as the most frequent tool users were adult and juvenile males. In contrast, females demonstrated a higher frequency of simple manipulations (Table 7). Furthermore, in addition to being the most frequent manipulators, males were more effective at acquiring the food item following the food processing.

Adult male capuchin monkeys are generally larger than adult females and tend to manipulate heavier objects, such as stones (Spagnoletti et al. 2011), which is consistent with the tool use observed in the BNP. Given that most manipulations observed at the BNP involved the use of stones as objects (approximately 45%, Table 4) and the executed actions primarily consisted of “hitting” (approximately 88%, Table 4), it is expected that individuals with greater body strength would have an advantage in these specific manipulations.

Conversely, adult females spend considerable time foraging in garbage bins and interacting with humans (Table 7), strategies that may yield higher success rates than manipulating natural items. Since females incur higher reproductive costs, they may be more sensitive to the energetic payoffs of foraging strategies, as the energetic loss resulting from failure in complex manipulations is more detrimental to them (Spagnoletti et al. 2011).

The observational method employed, the “all occurrences” method (Altmann 1974), can be potentially biased by the observer, as well as by variables such as the animal’s personality or the ease with which the capuchin can be observed. Since these variables were not adequately controlled during observation, it is possible that males were simply observed more frequently than females, and this bias may be a subproduct of the observation method. Despite this, the capuchin monkeys at the site were highly habituated to human contact, and even females with infants would approach the observer at short distances (Figure S1, Supplementary Material).

Tool use was strongly correlated with other forms of manipulation, supporting the idea that simpler manipulations act as training for more complex ones, a finding consistent with Koops et al. (2015). The ranking of individuals who most frequently cracked nuts was essentially the same as the ranking of individuals who performed the most manipulations in other ways (simple manipulation and proto-tools), except for JM1 and JF1, who exchanged ranks in tool use (Table 04).

Food acquisition (i.e., obtaining food at the conclusion of the manipulation event) occurred more frequently in food processing that did not involve tool use and when the individuals were male. These outcomes do not entirely align with our initial expectations. Palm nut cracking is a notably energy-intensive activity (Mangalam and Fragaszy 2015), increasing the diet quality of capuchin monkeys (Izar et al. 2022), therefore expected to be maintained with considerable efficiency (Visalberghi et al. 2021). Despite being infrequent, palm nut cracking has persisted in our study group for at least fifteen years (Waga et al. 2006), indicating that the individuals engaging in the behavior are not novices. Consequently, a higher level of proficiency in this particular behavior would have been anticipated.

Our data suggests that individuals who frequently interacted with humans and foraged in refuse (garbage) exhibited a higher frequency of simpler manipulative behaviors. Specifically, a greater frequency of both simple manipulation and proto-tool use was associated with increased refuse foraging. A positive relationship was also found between the frequency of simple manipulation and human interaction. Importantly, however, these anthropogenic variables did not appear to influence the frequency of complex tool use. Approximately 56% of the objects used as proto-tools were anthropogenic items (Table 5), indicating that simpler manipulations frequently targeted anthropogenic objects and, consequently, were more subject to anthropogenic influence. The fact that human objects are often designed to fit human hands (e.g., soda cans) may have stimulated the manipulation of these items by the capuchin monkeys (Dhananjaya et al. 2022).

A further point is that a large proportion of the capuchin monkeys’ energetic returns are derived from packaged food items found in refuse (Camargo et al. 2024). Crucially, this resource is available to the monkeys year-round, unlike natural resources such as palm nuts, which are seasonal. Therefore, the animals may have greater customary experience manipulating refuse items and consequently exhibit higher proficiency in handling these objects (Mangalam and Singh 2013).

Contrary to expectations, the adult male Ra and adult females Co and Ri were seldom observed engaging in palm nut cracking. This is an unexpected finding, as it is generally assumed that adult individuals, particularly males, perform most of the palm nut cracking due to their proficiency, size, and strength, a pattern observed in other capuchin monkeys and primates (e.g. Moura and Lee 2010; Spagnoletti et al. 2011). Indeed, Ra, Co, and Ri were among the most active individuals in seeking anthropogenic food. Co and Ri frequently employed aggressive strategies to obtain food directly from visitors, often with Ra acting in a defensive capacity (Camargo et al. 2024; Lousa et al. 2024). This suggests that these individuals prioritized alternative foraging strategies over tool use. These strategies for accessing anthropogenic food provided a reliable source of energy-rich resources, which satisfied their dietary needs (Camargo et al. 2024) and, consequently, diminished the motivation for engaging in alternative foraging strategies, such as tool use. Consistent with the findings of Roncero et al. (2023), access to anthropogenic food may be a contributing factor to the diminishing complexity of the population’s manipulative behaviors.

However, a contrasting pattern was observed in two other adult males, Mi and Di, who were also among the individuals most active in human-related foraging and garbage consumption. Notably, these same individuals were also the most frequent tool users. This finding indicates that these foraging strategies are not necessarily mutually exclusive.

Some manipulative behaviors, while not classified as tool use, can be considered complex. In urban environments, monkeys are frequently observed engaging in manipulative behaviors with anthropogenic objects that differ significantly from those seen in wild settings (Pal et al. 2022; Bender 2024). For example, in the current study, we observed monkeys using human packaging as containers to transport food. In other locations, capuchin monkeys have been anecdotally observed opening backpacks or unscrewing bottles, which may involve complex bimanual behaviors (Leca et al. 2011). Within the BNP, capuchins have also been documented breaching Styrofoam boxes and violating food packaging, behaviors that involve complex interactions with anthropogenic objects. This manipulative capacity is also employed during interactions with humans, which can lead to human-wildlife coexistence issues (Camargo et al. 2024). This aspect of their behavior warrants more thorough investigation in future studies, as new research on object manipulation and tool use by urban capuchin monkeys is necessary. Above all, more data on other manipulative activities not related to palm nut cracking is fundamental, as the vast majority of studies focus on this behavior.

## Supplementary Information

Below is the link to the electronic supplementary material.

Supplementary Material 1

## References

[CR1] Aguiar LM, Cardoso RM, Back JP, Carneiro EC, Suzin A, Ottoni EB (2014) Tool use in urban populations of capuchin monkeys *Sapajus* spp. (Primates: Cebidae). Zoologia 31: 516–519. 10.1590/S1984-46702014000500012

[CR2] Albuquerque Freire L, Cavalcante RS, Raimundo RL, de Castro CS, Izar P (2025) Household waste: a hidden threat to capuchin monkeys (*Sapajus libidinosus*) in the Caatinga, Pernambuco, Northeastern Brazil. Int J Primatol 46(2):306–310. 10.1007/s10764-024-00474-y

[CR3] Altmann J (1974) Observational study of behavior: sampling methods. Behaviour 49:227–266. 10.1163/156853974X00534 4597405 10.1163/156853974x00534

[CR4] Anca ED, Wallis J (2024) Plastic pollution and human–primate interactions: a growing conservation concern. Camb Prism Plast e10. 210.1017/plc.2024.10

[CR6] Barros RM et al (2025) Electrocutions in free-ranging platyrrhine nonhuman primates: diagnostic features for a threatening condition. Am J Primatol 87:e70039. 10.1002/ajp.70039 40253702 10.1002/ajp.70039 PMC12009613

[CR5] Bates D, Mächler M, Bolker B, Walker S (2015) Fitting linear mixed-effects models using lme4. J Stat Softw 67:1–48. 10.18637/jss.v067.i01

[CR7] Bender D (2024) Manipulação de objetos e uso de ferramentas em macacos-prego urbanos (*Sapajus* sp.)

[CR8] Camargo MR, Lousa TC, Mota RV, Mendes FD (2024) Interactions with humans reduce the success of foraging for anthropogenic food by capuchin monkeys (*Sapajus libidinosus*) in Brasília National Park, Brazil. Am J Primatol 86:e23620. 10.1002/ajp.23620 38506254 10.1002/ajp.23620

[CR9] Cardoso RM, Ottoni EB (2016) The effects of tradition on problem solving by two wild populations of bearded capuchin monkeys in a probing task. Biol Lett 12:20160604. 10.1098/rsbl.2016.0604 27881763 10.1098/rsbl.2016.0604 PMC5134038

[CR10] Coelho CG, Falótico T, Izar P et al (2015) Social learning strategies for nut-cracking by tufted capuchin monkeys (Sapajus spp). Anim Cogn 18:911–919. 10.1007/s10071-015-0861-5 25800169 10.1007/s10071-015-0861-5

[CR11] Corat C, Siqueira J, Ottoni EB (2015) Sequential organization and optimization of the nut-cracking behavior of semi-free tufted capuchin monkeys (Sapajus sp). Primates 57:113–121. 10.1007/s10329-015-0491-1 26411435 10.1007/s10329-015-0491-1

[CR22] de la García A, Oklander LI, Kowalewski MM, Fernandez-Duque E (2023) Human and non-human primate coexistence in argentina: conflicts and solutions. Animals 13:3331. 10.3390/ani13213331 37958086 10.3390/ani13213331 PMC10648367

[CR52] de Resende BD, Ottoni EB, Fragaszy DM (2008) Ontogeny of manipulative behavior and nut-cracking in young tufted capuchin monkeys (Cebus apella): a perception-action perspective. Dev Sci 11:828–840. 10.1111/j.1467-7687.2008.00731.x 19046151 10.1111/j.1467-7687.2008.00731.x

[CR12] Devaux CA, Mediannikov O, Medkour H, Raoult D (2019) Infectious disease risk across the growing human-non human primate interface: a review of the evidence. Front Public Health 7:305. 10.3389/fpubh.2019.00305 31828053 10.3389/fpubh.2019.00305 PMC6849485

[CR13] Dhananjaya T, Das S, Harpalani M, Huffman MA, Singh M (2022) Can urbanization accentuate hand use in the foraging activities of primates? Am J Biol Anthropol 178:667–677. 10.1002/ajpa.24532 36790685 10.1002/ajpa.24532

[CR16] Falótico T, Ottoni EB (2013) Stone throwing as a sexual display in wild female bearded capuchin monkeys, Sapajus libidinosus. PLoS ONE 8:e79535. 10.1371/journal.pone.0079535 24278147 10.1371/journal.pone.0079535 PMC3836890

[CR17] Falótico T, Ottoni EB (2014) Sexual bias in probe tool manufacture and use by wild bearded capuchin monkeys. Behav Process 108:117–122. 10.1016/j.beproc.2014.09.036 10.1016/j.beproc.2014.09.036 25446625

[CR18] Falótico T, Ottoni EB (2016) The manifold use of pounding stone tools by wild capuchin monkeys of Serra Da Capivara National Park, Brazil. Behaviour 153:421–442. 10.1163/1568539X-00003357

[CR20] Falótico T, Ottoni EB (2023) Greater tool use diversity is associated with increased terrestriality in wild capuchin monkeys. Am J Biol Anthropol 181:312–317. 10.1002/ajpa.24740 37067352 10.1002/ajpa.24740

[CR15] Falótico T, Labruna MB, Verderane MP, De Resende BD, Izar P, Ottoni EB (2007) Repellent efficacy of formic acid and the abdominal secretion of carpenter ants (Hymenoptera: Formicidae) against amblyomma ticks (Acari: Ixodidae). J Med Entomol 44:718–721. 10.1093/jmedent/44.4.718 17695031 10.1603/0022-2585(2007)44[718:reofaa]2.0.co;2

[CR19] Falótico T, Siqueira JO, Ottoni EB (2017) Digging up food: excavation stone tool use by wild capuchin monkeys. Sci Rep 7:6278. 10.1038/s41598-017-06541-0 28740211 10.1038/s41598-017-06541-0 PMC5524703

[CR14] Falótico T, Bueno CQ, Ottoni EB (2021) Ontogeny and sex differences in object manipulation and probe tool use by wild tufted capuchin monkeys (Sapajus libidinosus). Am J Primatol 83:e23251. 10.1002/ajp.23251 33666265 10.1002/ajp.23251

[CR21] Fragaszy DM, Aiempichitkijkarn N, Eshchar Y, Mangalam M, Izar P, Resende B, Visalberghi E (2023) The development of expertise at cracking palm nuts by wild bearded capuchin monkeys, Sapajus libidinosus. Anim Behav 197:1–14. 10.1016/j.anbehav.2022.12.008

[CR71] García de la Chica A, Oklander LI, Kowalewski MM, Fernandez-Duque E (2023) Human and non-human primate coexistence in Argentina: Conflicts and solutions. Animals 13(21):3331. 10.3390/ani13213331 10.3390/ani13213331 PMC10648367 37958086

[CR23] Goldsborough Z, Crofoot MC, Barrett BJ (2024) Male-biased stone tool use by wild white‐faced capuchins (*Cebus capucinus imitator*). Am J Primatol 86:e23594. 10.1002/ajp.23594 38196199 10.1002/ajp.23594

[CR24] Gutierres JS, Pereira FSM, Lynch JW, Vidotto Magnoni AP (2025) Stone tool use by Black-Horned capuchin monkeys (Sapajus nigritus cucullatus) in an urban park in Londrina, Brazil. Am J Primatol 87:e23704. 10.1002/ajp.23704 10.1002/ajp.23704 39749686

[CR25] Hartig F (2024) _DHARMa: residual diagnostics for hierarchical (multi-level/mixed) regression models 10.32614/CRAN.package.DHARMa

[CR26] Haslam M, Luncz LV, Staff RA, Bradshaw F, Ottoni EB, Falótico T (2016) Pre-Columbian monkey tools. Curr Biol 26:R521–R522. 10.1016/j.cub.2016.05.046 27404235 10.1016/j.cub.2016.05.046

[CR72] Hayashi M (2015) Perspectives on object manipulation and action grammar for percussive actions in primates. Philos Trans R Soc B 370(1682):20140350. 10.1098/rstb.2014.0350 10.1098/rstb.2014.0350 PMC4614713 26483528

[CR27] Heldstab SA, Kosonen ZK, Koski SE, Burkart JM, van Schaik CP, Isler K (2016) Manipulation complexity in primates coevolved with brain size and terrestriality. Sci Rep 6:24528. 10.1038/srep24528 27075921 10.1038/srep24528 PMC4830942

[CR28] Horowitz C, Martins CR, Walter BMT (2013) Flora exótica no Parque Nacional de Brasília: Levantamento e classificação Das espécies. Biodivers Bras 3:50–73. 10.37002/biodiversidadebrasileira.v3i2.353

[CR29] Izar P et al (2022) Stone tools improve diet quality in wild monkeys. Curr Biol 32:4088–4092. 10.1016/j.cub.2022.07.056 35985326 10.1016/j.cub.2022.07.056

[CR30] Jaman MF, Huffman MA (2013) The effect of urban and rural habitats and resource type on activity budgets of commensal rhesus macaques (*Macaca mulatta*) in Bangladesh. Primates 54:49–59. 10.1007/s10329-012-0330-6 22987063 10.1007/s10329-012-0330-6

[CR31] Kaburu SS, Marty PR, Beisner B, Balasubramaniam KN, Bliss-Moreau E, Kaur K et al (2019) Rates of human–macaque interactions affect grooming behavior among urban‐dwelling rhesus macaques (Macaca mulatta). Am J Biol Anthropol 168:92–103. 10.1002/ajpa.23722 10.1002/ajpa.23722 30368773

[CR32] Koops K, Furuichi T, Hashimoto C, Van Schaik CP (2015) Sex differences in object manipulation in wild immature chimpanzees (*Pan troglodytes schweinfurthii*) and bonobos (*Pan paniscus*): Preparation for tool use? Plos one. 10:e0139909. 10.1371/journal.pone.0139909 10.1371/journal.pone.0139909 PMC4596577 26444011

[CR33] Laird MF et al (2022) Feeding postural behaviors and food geometric and material properties in bearded capuchin monkeys (*Sapajus libidinosus*). Am J Biol Anthropol 178:3–16. 10.1002/ajpa.24501

[CR34] Leca JB, Gunst N, Huffman M (2011) Complexity in object manipulation by Japanese macaques (Macaca fuscata): a cross-sectional analysis of manual coordination in stone handling patterns. J Comp Psychol 125(1):61 21244141 10.1037/a0020868

[CR35] Lee VE, Thornton A (2021) Animal cognition in an urbanised world. Front Ecol Evol 9:633947 34409044 10.3389/fevo.2021.633947 PMC7611524

[CR77] Lee VE, Thornton A (2021) Animal cognition in an urbanised world. Front Eco and Evol 9:633947. 10.3389/fevo.2021.633947 10.3389/fevo.2021.633947 PMC7611524 34409044

[CR36] Lenth R (2025) _emmeans: Estimated Marginal Means, aka Least-Squares Means_. 10.32614/CRAN.package.emmeans

[CR38] Lousa TC, de Grande TO, Mendes FDC (2022) Time budget and foraging strategies of two provisioned groups of tufted capuchin monkeys, *Sapajus libidinosus*, in a small, seasonal urban forest fragment. Primates 63:387–395. 10.1007/s10329-022-00993-3 35599294 10.1007/s10329-022-00993-3

[CR37] Lousa TC, Camargo MR, Mota RV, Mendes FDC (2024) Disputes over feeding resources provoke agonistic interactions between capuchins and humans in Brasília National park. Brazil Anthrozoös 37:1051–1065. 10.1080/08927936.2024.2412407

[CR39] Mangalam M, Fragaszy DM (2015) Wild bearded capuchin monkeys crack nuts dexterously. Curr Biol 25:1334–1339. 10.1016/j.cub.2015.03.035 25936553 10.1016/j.cub.2015.03.035

[CR40] Mangalam M, Singh M (2013) Flexibility in food extraction techniques in urban free-ranging Bonnet macaques, Macaca radiata. PLoS ONE 8:e85497. 10.1371/journal.pone.0085497 24376883 10.1371/journal.pone.0085497 PMC3869890

[CR41] Mannu M, Ottoni EB (2009) The enhanced tool-kit of two groups of wild bearded capuchin monkeys in the caatinga: tool making, associative use, and secondary tools. Am J Primatol 71:242–251. 10.1002/ajp.20642 19051323 10.1002/ajp.20642

[CR74] Mendes FD, Cardoso RM, Ottoni EB, Izar P, Villar DN, Marquezan RF (2015) Diversity of nutcracking tool sites used by Sapajus libidinosus in Brazilian Cerrado. American J Prima 77(5):535–546. 10.1002/ajp.22373 10.1002/ajp.22373 25676549

[CR42] Mota RV (2018) Análise etológica do forrageio social de alimentos antrópicos por Sapajus libidinosus (Spix, 1823) (Primates: Cebidae). Dissertation on Behavioral Sciencies. Available via DIALOG. https://cdc.unb.br/wp-content/uploads/2024/09/Tese_Ricardo_Vasquez26Dez2018_LowRes.pdf

[CR43] Moura AC, Lee PC (2010) Wild capuchins show male-biased feeding tool use. Int J Primatol 31:457–470. 10.1007/s10764-010-9406-6

[CR73] Filho MR, Camargo MR, Mendes FD (2021) Male-directed object use by proceptive female bearded capuchin monkeys (Sapajus libidinosus) in captivity. Inter J Primatology 42(2):187–200. 10.1007/s10764-020-00195-y

[CR44] Ottoni EB (2015) Tool use traditions in nonhuman primates: the case of tufted capuchin monkeys. Hum Ethol Bull 30:22–40

[CR45] Ottoni EB, Izar P (2008) Capuchin monkey tool use: overview and implications. Evol Anthropol 17:171–178. 10.1002/evan.20185

[CR46] Ottoni EB, Mannu M (2001) Semifree-ranging tufted capuchins (Cebus apella) spontaneously use tools to crack open nuts. Int J Primatol 22:347–358. 10.1023/a:1010747426841

[CR47] Ottoni EB, de Resende BD, Izar P (2005) Watching the best nutcrackers: what capuchin monkeys (Cebus apella) know about others’ tool-using skills. Anim Cogn 8:215–219. 10.1007/s10071-004-0245-8 15719240 10.1007/s10071-004-0245-8

[CR48] Pal A, Mahato S, Leca JB, Sinha A (2022) Blowing the lid off! Bottle-directed, extractive foraging strategies in synurbic Bonnet macaques *Macaca radiata* in Southern India. Front Psychol 13:973566. 10.3389/fpsyg.2022.973566 36755978 10.3389/fpsyg.2022.973566 PMC9900441

[CR49] Parker ST, Gibson KR (1977) Object manipulation, tool use and sensorimotor intelligence as feeding adaptations in Cebus monkeys and great apes. J Hum Evol 6(7):623–641

[CR50] Praill LC et al (2023) Road infrastructure and primate conservation: introducing the global primate roadkill database. Animals 13:1692. 10.3390/ani13101692 37238122 10.3390/ani13101692 PMC10215684

[CR51] Reader SM, Laland KN (2001) Primate innovation: sex, age and social rank differences. Int J Primatol 22:787–805. 10.1023/A:1012069500899

[CR53] Roncero P, de Mendonca-Furtado O, Izar P (2023) Human-induced rapid environmental change: a case study showing negative impact on animal culture. J Nat Conserv 74:126424. 10.1016/j.jnc.2023.126424

[CR54] Ruas RB, Costa LMS, Bered F (2022) Urbanization driving changes in plant species and communities–A global view. Glob Ecol Conserv 38:e02243. 10.1016/j.gecco.2022.e02243

[CR55] Sabbatini G, Stammati M, Tavares MCH, Giuliani M, Visalberghi E (2006) Interactions between humans and capuchin monkeys (Cebus libidinosus) in the Parque Nacional de Brasília, Brazil. Appl Anim Behav Sci 97:272–283. 10.1016/j.applanim.2005.07.002

[CR56] Sacramento TS, Mendes FDC, Peres MK, Tavares MCH (2017) Comportamento alimentar e padrão de atividades de macacos-prego (*Sapajus libidinosus*) Aprovisionados no Parque Nacional de Brasília, DF. In: Oliveira MAB, Ferreira RG, da Luna V (eds) A primatologia no Brasil, vol 14. rd edn. Recife, pp 158–172

[CR57] Shumaker RW, Walkup KR, Beck BB (2024) Animal tool behavior: the use and manufacture of tools by animals. JHU, Baltimore

[CR58] Spagnoletti N, Visalberghi E, Ottoni E, Izar P, Fragaszy D (2011) Stone tool use by adult wild bearded capuchin monkeys (Cebus libidinosus). Frequency, efficiency and tool selectivity. J Hum Evol 61:97–107. 10.1016/j.jhevol.2011.02.010 21470663 10.1016/j.jhevol.2011.02.010

[CR59] Taffoni F, Polizzi di Sorrentino E, Sabbatini G, Formica D, Truppa V (2017) Primates’ propensity to explore objects: How manual actions affect learning in children and capuchin monkeys. In: Bertolaso M., Di Stefano N. (eds) The Hand. Studies in Applied Philosophy, Epistemology and Rational Ethics, vol 38. Gewerbestrasse, pp 55–73. 10.1007/978-3-319-66881-9_4

[CR60] Thatcher HR, Downs CT, Koyama NF (2021) The costs of urban living: human–wildlife interactions increase parasite risk and self-directed behaviour in urban Vervet monkeys. J Urban Ecol 7:juab031. 10.1093/jue/juab031

[CR61] Thatcher HR, Downs CT, Koyama NF (2023) Primates in the urban mosaic: Terminology, flexibility, and management. Primates in anthropogenic landscapes: exploring primate behavioural flexibility across human contexts. Springer International Publishing, Cham, pp 121–137. 10.1007/978-3-031-11736-7_8

[CR70] Thacher JD, Oudin A, Flanagan E, Mattisson K, Albin M, Roswall N, Pyko A, Aasvang GM, Andersen ZJ, Borgquist S, Brandt J, Broberg K, Cole-Hunter T, Eriksson C, Eneroth K, Gudjonsdottir H, Helte E, Ketzel M, Lanki T, Lim YH, Leander K, Ljungman P, Manjer J, Männistö S, Raaschou-Nielsen O, Pershagen G, Rizzuto D, Sandsveden M, Selander J, Simonsen MK, Stucki L, Spanne M, Stockfelt L, Tjønneland A, Yli-Tuomi T, Tiittanen P, Valencia VH, Ögren M, Åkesson A, Sørensen M (2023) Exposure to long-term source-specific transportation noise and incident breast cancer: A pooled study of eight Nordic cohorts. Envir Inter 178:108108. 10.1016/j.envint.2023.108108 10.1016/j.envint.2023.108108 37490787

[CR62] Torres-Romero EJ, Nijman V, Fernández D, Eppley TM (2023) Human‐modified landscapes driving the global primate extinction crisis. Glob Chang Biol 29:5775–5787. 10.1111/gcb.16902 37578114 10.1111/gcb.16902

[CR75] Torigoe T (1985) Comparison of object manipulation among 74 species of non-human primates. Primates 26(2):182–194. 10.1007/BF02382017

[CR63] Truppa V, Carducci P, Sabbatini G (2019) Object grasping and manipulation in capuchin monkeys (genera Cebus and Sapajus). Biol J Linn Soc 127:563–582. 10.1093/biolinnean/bly131

[CR64] United Nations (2024) Cities and Climate Action: World Cities Report 2024. https://unhabitat.org/wcr/

[CR65] Verderane MP, Falótico T, Resende BD, Labruna MB, Izar P, Ottoni EB (2007) Anting in a semifree-ranging group of Cebus Apella. Int J Primatol 28:47–53. 10.1007/s10764-006-9102-8

[CR67] Visalberghi E, Fragaszy DM, Izar P, Ottoni EB (2005) Terrestriality and tool use. Science 308:951–952. 10.1126/science.308.5724.951c 15890860 10.1126/science.308.5724.951c

[CR68] Visalberghi E, Sabbatini G, Taylor AH, Hunt GR (2017) Cognitive insights from tool use in nonhuman animals. In: Call J, Burghardt GM, Pepperberg IM, Snowdon CT, Zentall T (eds) APA handbook of comparative psychology: Perception, learning, and cognition. American Psychological Association, Columbia, pp 673–701. 10.1037/0000012-030

[CR66] Visalberghi E, Barca V, Izar P, Fragaszy D, Truppa V (2021) Optional tool use: the case of wild bearded capuchins (Sapajus libidinosus) cracking cashew nuts by biting or by using percussors. Am J Primatol 83:e23221. 10.1002/ajp.23221 33300618 10.1002/ajp.23221

[CR69] Waga IC, Dacier AK, Pinha PS, Tavares MCH (2006) Spontaneous tool use by wild capuchin monkeys (Cebus libidinosus) in the Cerrado. Folia Primatol 77:337–344. 10.1159/000093698 10.1159/000093698 16912501

