# Supplementary material for: Object and tool manipulation diversity in an urban capuchin monkey (Sapajus libidinosus) group in Brasília National park
Source: Primates. 2026 Mar 7;67(3):347–59. doi: 10.1007/s10329-026-01242-7 (PMC13124907; doi:10.1007/s10329-026-01242-7)
Supplement: Supplementary file 1 — Supplementary Material 1 [file 10329_2026_1242_MOESM1_ESM.docx]

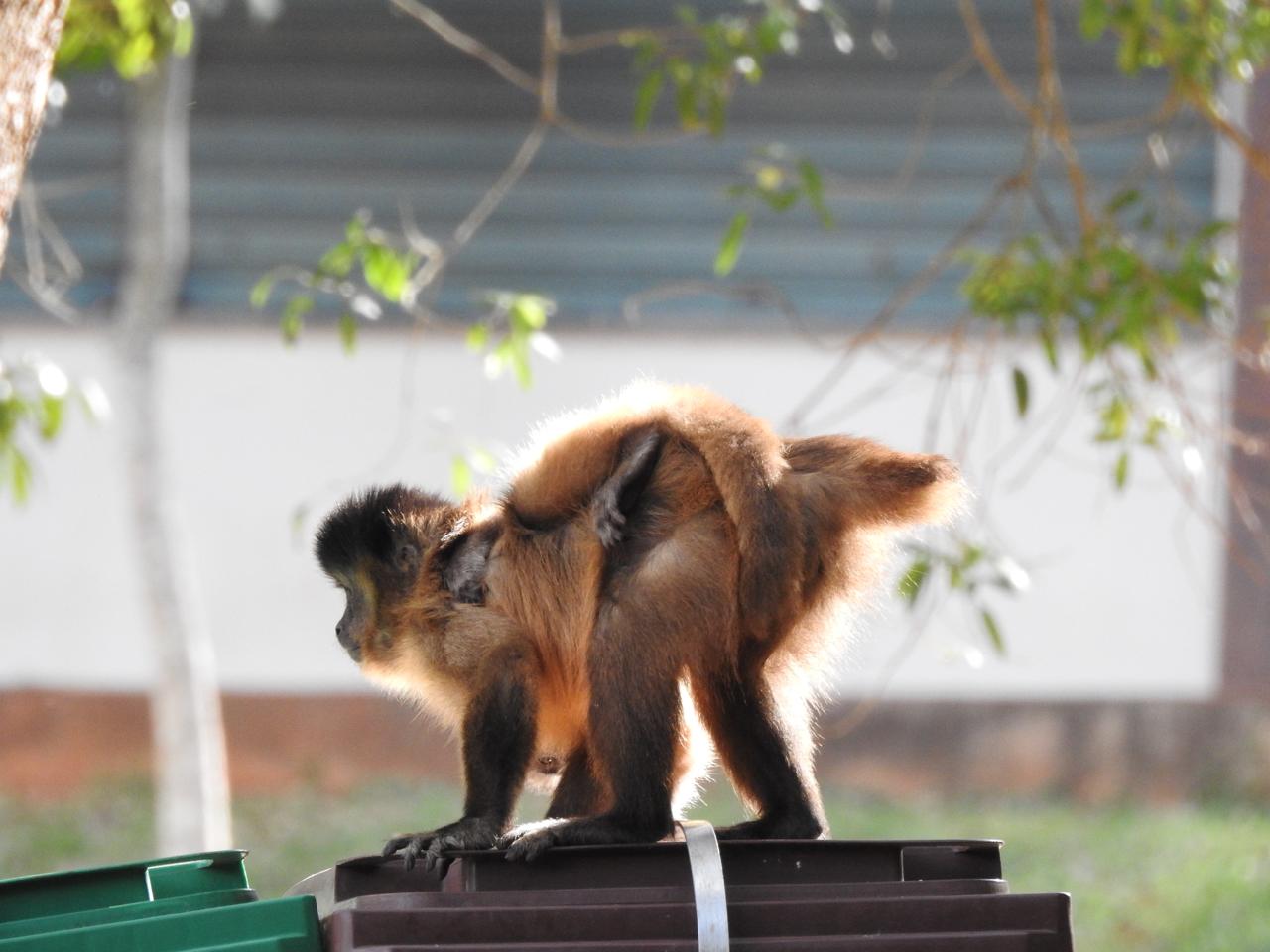

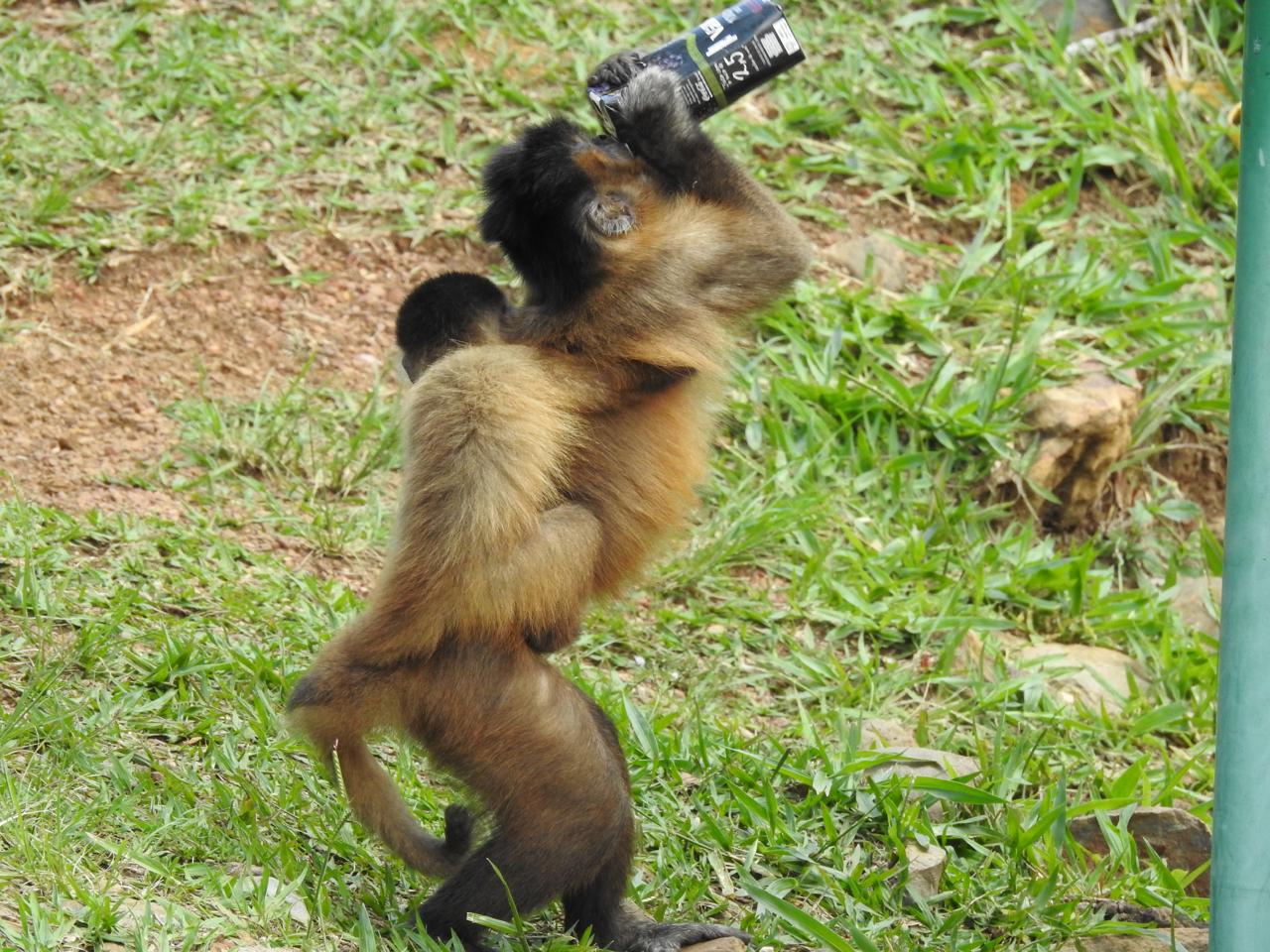

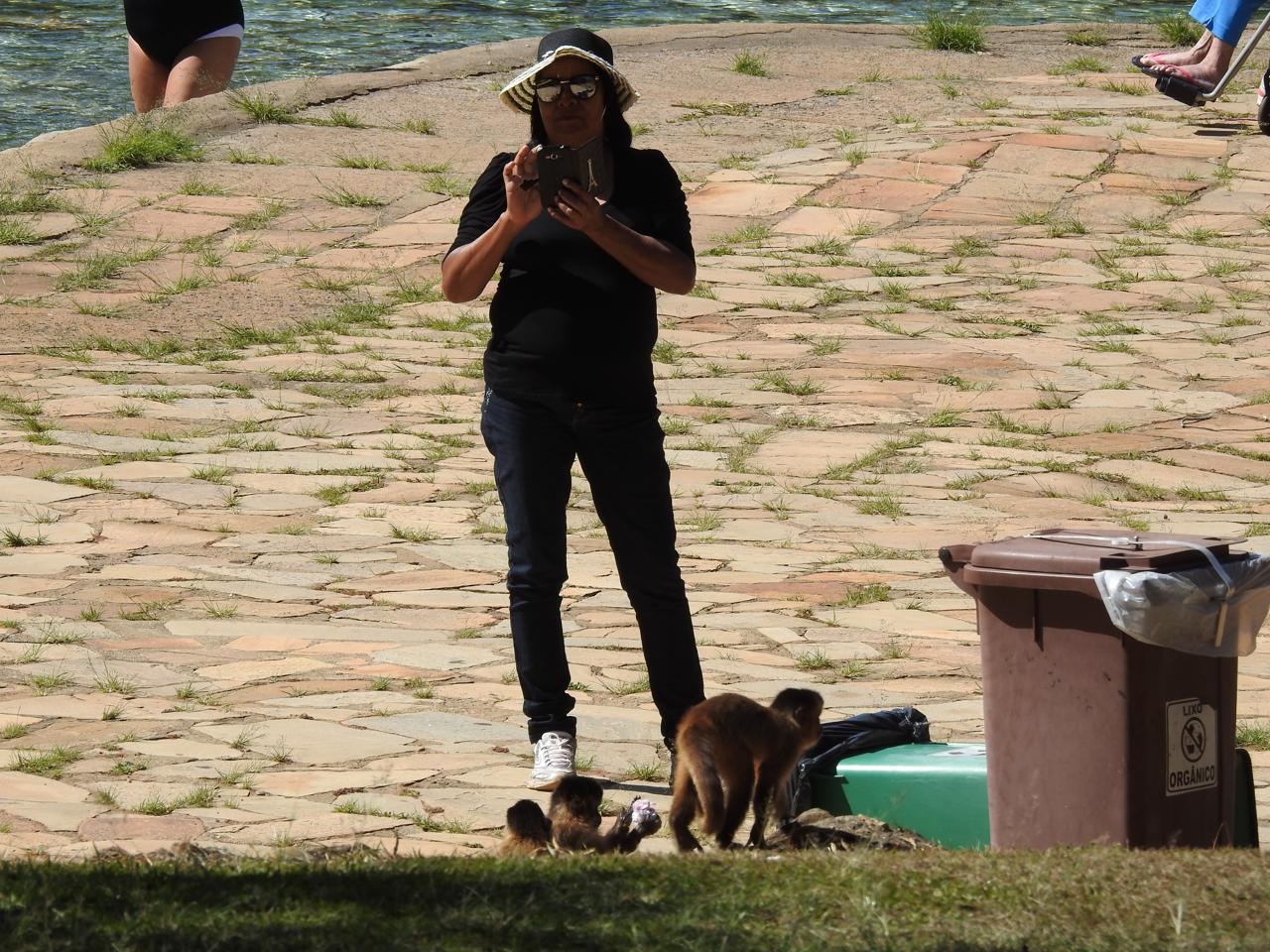


Figure S1. Images of adult females with dependent infants foraging in garbage and in close proximity to humans.
